# External Application of a Nomogram to Predict Survival and Benefit of Peripheral Blood Inflammatory Indexes in Limited-Stage Small Cell Lung Cancer

**DOI:** 10.3389/fonc.2022.873367

**Published:** 2022-05-11

**Authors:** Lijuan Wei, Qing Hou, Jianting Liu, Ningning Yao, Yu Liang, Xin Cao, Bochen Sun, Hongwei Li, Shuming Xu, Jianzhong Cao

**Affiliations:** ^1^ Department of Radiotherapy Center, Shanxi Province Cancer Hospital/Shanxi Hospital Affiliated to Cancer Hospital, Chinese Academy of Medical Sciences/Cancer Hospital Affiliated to Shanxi Medical University, Taiyuan, China; ^2^ Department of Radiology, Shanxi Children’s Hospital, Taiyuan, China

**Keywords:** small cell lung cancer, immune and inflammatory, prognoses prediction, overall survival, external application

## Abstract

**Background:**

Qi et al. recently proposed a nomogram to reveal the prognostic value of peripheral blood inflammatory indexes (named Risk) and predict overall survival (OS) in limited-stage small cell lung cancer (LS-SCLC). However, it hasn’t undergone external application so far. This study aimed to verify the role of Risk as a prognostic variable of OS and apply the nomogram externally.

**Methods:**

We used a retrospective analysis of clinical data of 254 patients diagnosed as LS-SCLC in Shanxi Cancer Hospital from January 2015 to December 2018 to apply Qi’s nomogram externally. We also performed subgroup analysis to explore the predictive value of Risk. The model was evaluated in terms of discrimination (the area under the ROC curve (AUC ROC) and calibration (calibration plots).

**Results:**

The prognosis of patients with low-Risk was significantly better than those with high-Risk in our cohort (p<0.01). The AUC of 1-, 2-, and 3-year OS was 0.644, 0.666, and 0.635, respectively. The calibration curve showed a nearly ideal calibration-slope of 1-, 2-, and 3-year OS (1.00 (0.41-1.59), 1.00 (0.54-1.46) and 1.00 (0.43-1.57), respectively).

**Conclusion:**

The external application of nomogram added Risk for predicting OS in LS-SCLC patients showed a moderate-to-good performance using a cohort with different case-mix characteristics. The external application confirmed the predictive value of Risk and the usefulness of the nomogram for the prediction of OS.

## Introduction

Lung cancer remains the leading cause of cancer mortality in China and worldwide (1, 2). Small cell lung cancer (SCLC), accounting for approximately 15% of all lung cancer, has a poor prognosis because of its very aggressive clinical course and early metastasis. Limited stage small cell lung cancer (LS-SCLC) makes up almost 40%, with a median overall survival (OS) of 20 months (3, 4). The early accurate prediction of patients’ prognoses is significant for making the most favorable treatment decisions. At present, the standard treatment for LS-SCLC is the combination of chemotherapy, thoracic radiotherapy, and prophylactic cranial irradiation (PCI) (3). Although immunotherapy has become the new standard of first-line treatment for extensive-stage SCLC (ES-SCLC), the additional benefit for LS-SCLC is not clear (3–5).

For years, cumulative studies have confirmed the predictive role of clinical variables on prognosis, remarkably immune and inflammatory response, which has been considered one of the main prognostic predictors in several solid tumors (6–9). Recently Qi et al. proposed a nomogram to estimate the additional benefit of Risk, an inflammation-related prognostic scoring system, in predicting prognosis for LS-SCLC (10). They found lower pretreatment neutrophil-to-lymphocyte ratio (NLR) and systemic inflammation index (SII) were significantly associated with better prognosis. In contrast, lower baseline platelet counts, lymphocyte counts, and albumin were indicators of worse OS. Further, they formulated Risk based on the optimal inflammation indexes by the LASSO-Cox model and grouped patients into low-Risk and high-Risk with “0” as a cutoff value. Compared with high-Risk, patients with low-Risk tend to have a longer survival time, with a median OS of 36.5 and 17.7 months (*P* < 0.001). Add Risk to nomogram, a remarkable improvement in predictive accuracy was observed compared with clinical factors alone. The Qi’s study showed that systemic inflammation index was a routine, low-cost, and readily available serum biomarker indicating poor prognosis, and the nomogram containing Risk could achieve a better prognosis prediction of LS-SCLC. However, it has not been externally applied so far.

This study aimed to perform an external application of Qi’s prediction model, verify the predictive value of Risk, and evaluate the nomogram’s applicability in a single center with different case-mix characteristics.

## Materials and Methods

### Patient Population

This retrospective observational external application cohort study was carried out in patients pathologically diagnosed as SCLC in Shanxi Cancer Hospital from January 2015 to December 2018. Inclusion criteria were similar to the original study (10). All patients have been reassessed as LS-SCLC by the Veterans Administration Lung Study Group (VALG) staging standard (2017 NCCN guidelines), and all patients received concurrent or sequential chemoradiotherapy. The cases analyzed in the study were complete, and we didn’t use padding techniques to deal with the missing data. We reviewed clinical data, treatment records, and follow-up through the electronic medical record system or by contacting the patients directly. The last follow-up ended on August 20, 2020. This study received ethical approval from the Ethics committee of Shanxi Provincial Cancer hospital (No. 202102). As this was a retrospective study and all information related to the patient’s identity was hidden, the Ethics Committee waived informed consent. This study follows the ethical criteria of the Declaration of Helsinki.

### Data Collection

The inflammatory variables included in Risk and relevant clinicopathological characteristics in the nomogram were collected. The calculation formulas of platelet-to-lymphocyte ratio (PLR), NLR, and SII were the same as those in the original study (10). The nomogram estimated the predicted probability of 1-, 2-, and 3-year OS rates by calculating the total points for each patient. The observed 1-, 2-, and 3-year OS rates were derived from follow-up data in our study cohort.

### Statistical Analyses

Baseline characteristics in Qi’s and external application cohorts were described as counts (n) and percentages and compared using the Chi-square test. A *P*-value of <0.05 was considered statistically significant. Replicate codes in the original study to construct Risk. The cutoff values of inflammatory variables were determined by the package “maxstat” of R software based on OS. The selection of optimal prognostic factors was performed by the package “glmnet” of R software. The multicollinearity among variables was assessed by variance inflation factors (VIFs). A cutoff value was adopted to dichotomize the total cohort into high-Risk and low-Risk groups. Differences in OS between Risk groups were estimated in the subgroup analysis. A *P* < 0.05 indicated statistical significant.

The total score for each patient was calculated based on the weight of each factor in the original nomogram and was used to depict the receiver-operating characteristics (ROC) curves. The Discrimination of the nomogram was evaluated by Harrell’s concordance index (C-index) or the area under the receiver-operating characteristics (AUROC) curves. The value of AUC ranged from 0.5 (random chance) to 1.0 (perfect discrimination), and higher AUC indicated higher prediction accuracy. Then the calibration curve was plotted to visually assess the calibration of the nomogram by comparing the predicted survival probabilities with the observed survival probabilities. A close to 45-degree calibration indicated a perfect prediction model.

All statistical analyses in this article were performed using R statistical software (version 3.6.3).

## Results

### Characteristics of Patients

This study included 254 LS-SCLC patients based on the inclusion and exclusion criteria. The median values of platelet, lymphocyte, albumin, NLR, PLR, and SII were 264 × 10^9^/L (range = 41-717 ×10^9^/L), 1.77× 10^9^/L (range = 0.59-4.02 × 10^9^/L), 42.5 × 10^9^g/L (range = 30-51.9 × 10^9^g/L), 2.52 (range = 0.7-22.68),159.99 (range = 18.14-590.16), and 686.49 (range = 57.69-8074.23), respectively. The median age was 60 years (range 28-76 years). The median follow-up time was 20.57 months, far less than the time in the original study (55.9 months). One hundred fifty-six deaths (61.4% of the 254 totals) had been observed during follow-up, similar to the original cohort (60.8%). The median OS was 22.2 months, less than the Qi’s cohort (25.7). The 1-, 2-, 3-year OS rates were 76.1%, 45.6% and 36.2%, respectively. **Table 1** shows the baseline characteristics of LS-SCLC patients in Qi’s and external application cohort. Compared with Qi’s cohort, patients in the external application cohort showed a higher proportion of males (82.7% vs. 72.8%, *P*=0.005) and smokers (77.2% vs. 69.5%, *P*=0.038). The percentages of both the T and N stages also had statistical differences(*P*<0.001). Overall, our patients tend to have more advanced T and N stages. The treatment methods varied the most. Our patients received less PCI (21.7% vs. 38.6%, *P*<0.001) and significantly more sequential chemoradiotherapy (73.2% vs. 19.8%, *P*<0.001) than Qi’s cohort.

**Table 1:** Baseline characteristics of the external application and Qi’s cohorts.

Variables	External application cohort	Qi’s cohort	*P* value
**gender**			0.005
Female	44 (17.3)	91 (27.2)	
Male	210 (82.7)	243 (72.8)	
**age**			0.614
<65	187 (73.6)	252 (75.4)	
>=65	67 (26.4)	82 (24.6)	
**smoke**			0.038
No	58 (22.8)	102 (30.5)	
Yes	196 (77.2)	232 (69.5)	
**KPS**			0.918
<80	38 (15.0)	51 (15.3)	
>=80	216 (85.0)	283 (84.7)	
**T stage**			< 0.001
1	28 (11.0)	40 (12)	
2	79 (31.1)	122 (36.5)	
3	46 (18.1)	102 (30.5)	
4	101 (39.8)	70 (21)	
**N stage**			< 0.001
0	33 (13.0)	32 (9.6)	
1	10 (3.9)	34 (10.2)	
2	114 (44.9)	194 (58.1)	
3	97 (38.2)	74 (22.2)	
**PCI**			< 0.001
No	199 (78.3)	205 (61.4)	
Yes	55 (21.7)	129 (38.6)	
**treatment**			< 0.001
Concurrent	68 (26.8)	268 (80.2)	
Sequential	186 (73.2)	66 (19.8)	

KPS, Karnofsky Performance Score; PCI, prophylactic cranial irradiation.

### Construction of Risk

**Figure 1:**
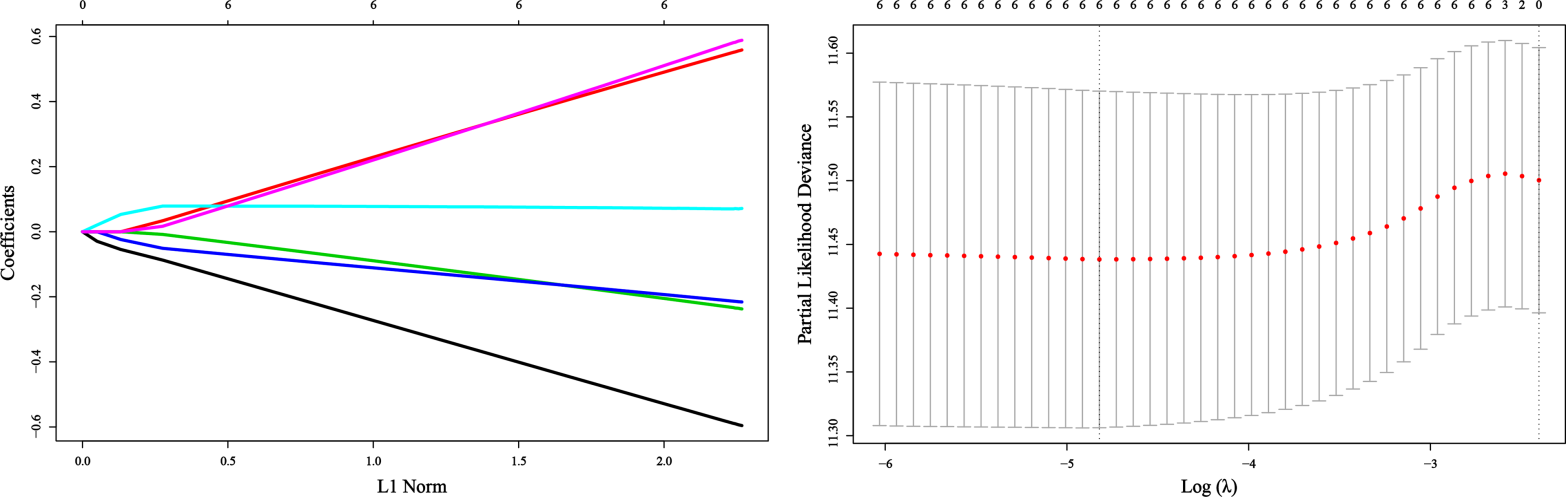
Construction of the Risk by the least absolute shrinkage and selection operator (LASSO) model in the application cohort.

Due to the different laboratory instruments used in the two hospitals, the range of reference values may be different. Varying the optimal cutoff value of each inflammatory variable is required. We copied the methods in the original article to determine the optimal cutoff values of these inflammatory markers, as shown in **Table 2**. We incorporated these variables into a least absolute shrinkage and selection operator (LASSO)-Cox regression model to define Risk’s prognostic scoring system (**Figure 1**). Risk = -0.5548*Platelet + 0.5171*Lymphocyte –0.2172*Albumin + 0.0715*NLR -0.2019*PLR +0.5411*SII. Consistent with the original article, all VIF values <5 (**Table 3**) indicate no collinearity among these variables. Using the cutoff value of 0.098 identified by Maxstat, we classified patients as low-Risk and high-Risk groups. The baseline characteristics in low-Risk and high-Risk were similar(all *P*>0.05), as shown in **Table 4**.

**Table 2:** The cutoff value of inflammation-related factors in external application cohorts.

Characteristics	Cutoff	Categories
PLT	265	High (≥265) vs. Low (<265)
LYM	2.42	High (≥2.42) vs. Low (<2.42)
NLR	2.87	High (≥2.87) vs. Low (<2.87)
PLR	91.58	High (≥91.58) vs. Low (<91.58)
SII	683.93	High (≥683.93) vs. Low (<683.93)
ALB	40.1	High (≥40.1) vs. Low (<40.1)

PLT, platelet; LYM, lymphocyte; NLR, neutrophil to lymphocyte ratio; PLR, platelet to lymphocyte ratio; SII, systemic inflammation index; ALB, albumin.

**Table 3:** VIF of inflammation-related factors in external application cohorts.

Variables	PLT	LYM	ALB	PLR	NLR	SII
**VIF**	1.587	1.432	1.072	1.446	1.842	2.457

VIF, variance inflation factors; PLT, platelet; LYM, lymphocyte; NLR, neutrophil to lymphocyte ratio; PLR, platelet to lymphocyte ratio; SII, systemic inflammation index; ALB, albumin.

**Table 4:** Baseline characteristics of low-risk group and high-risk group.

Characteristics	Total (n = 254)	Low-Risk (n = 187)	High-Risk (n = 57)	*P* value
**age**				0.724
<65	187	144 (73.1%)	43 (75.44%)	
≥65	67	53 (26.9%)	14 (24.56%)	
**gender**				0.124
Female	44	38 (19.29%)	6 (10.53%)	
Male	210	159 (80.71%)	51 (89.47%)	
**smoke**				0.47
No	58	47 (23.86%)	11 (19.3%)	
Yes	196	150 (76.14%)	51 (80.7%)	
**KPS**				0.059
≥80	216	172 (87.31%)	44 (77.19%)	
<80	38	25 (12.69%)	13 (22.81%)	
**PCI**				0.113
No	199	150 (76.14%)	49 (85.96%)	
Yes	55	47 (23.86%)	8 (14.04%)	
**treatment**				0.107
Concurrent	68	48 (24.37%)	20 (35.09%)	
Sequential	186	149 (75.63%)	37 (64.91%)	
**T stage**				0.463
1	28	24 (12.18%)	4 (7.02%)	
2	79	57 (28.93%)	22 (38.6%)	
3	46	36 (18.27%)	10 (17.54%)	
4	101	80 (40.61%)	21 (36.84%)	
**N stage**				0.494
0	33	23 (11.68%)	10 (17.54%)	
1	10	7 (3.55%)	3 (5.26%)	
2	114	92 (46.7%)	22 (38.6%)	
3	97	75 (38.07%)	22 (38.6%)	

KPS, Karnofsky Performance Score; PCI, prophylactic cranial irradiation.

### Application of Risk

**Figure 2:**
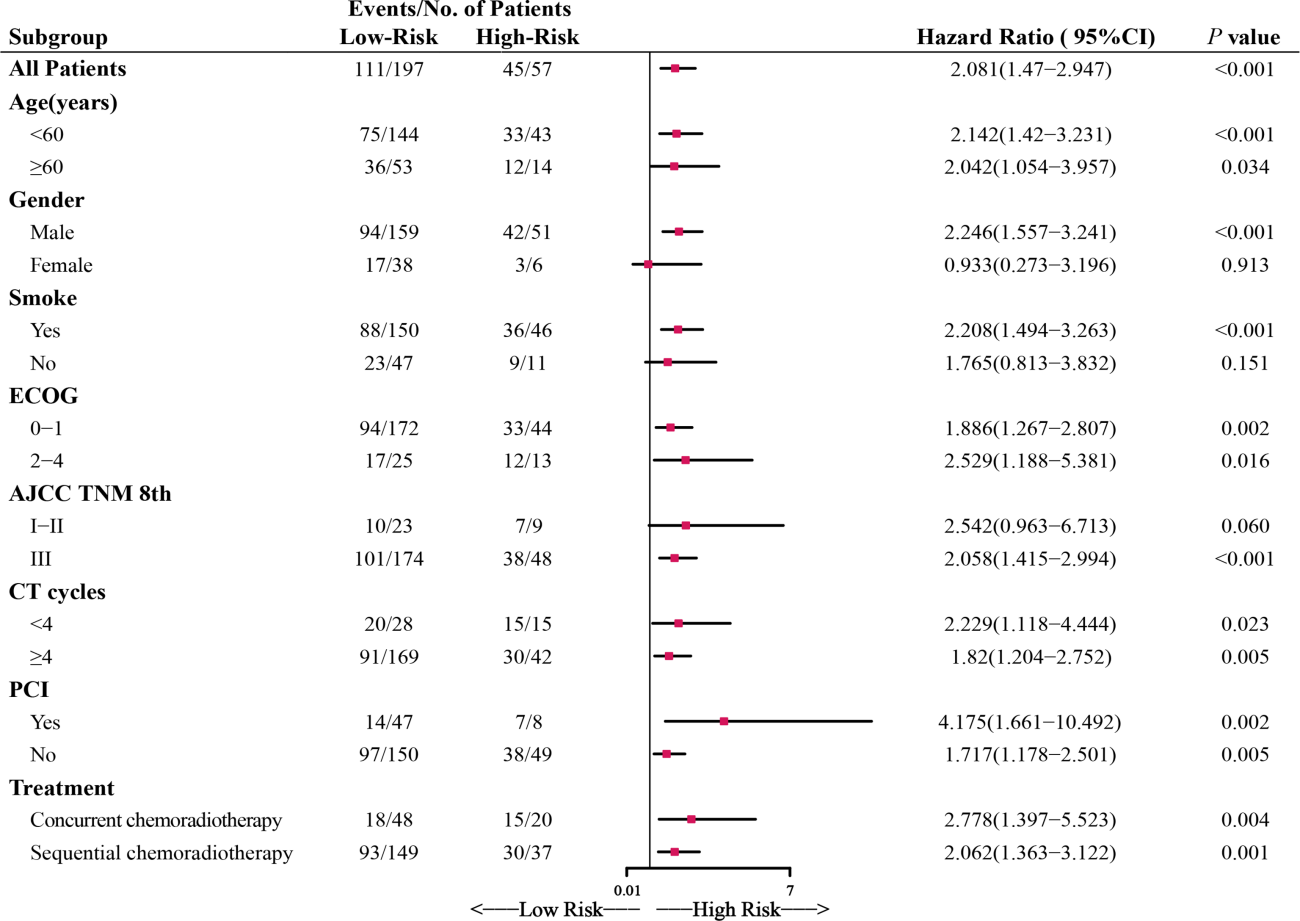
Forest plot depicting the hazard ratios (HRs) of low-Risk and high-Risk in the subgroup analysis of overall survival (OS). ECOG, Eastern Cooperative Oncology Group performance score; CT cycles, chemotherapy cycles; PCI, prophylactic cranial irradiation.

Compared with the high-Risk group, patients in the low-Risk group had significantly better prognoses. The low-Risk group had a 1-, 2- and 3-year OS of 82.0%, 51.9%, and 40.6%, while the high-Risk group had a 1-,2-,3- year OS of 55.4%, 23.9%, and 20.5%, respectively. Within the study period, a total of 156 deaths occurred, 111(59.4%) in the low-Risk group and 45(78.9%) in the high-Risk group. The subgroup analysis confirmed the good prognostic capacity of Risk (**Figure 2**). The forest plot showed each subgroup’s OS and HRs of low-Risk vs. high-Risk. The high-Risk group remained associated with poor prognosis in most subgroups except female and no-smoking, with the HRs ranging from 1.717 to 4.175(all *P*<0.05).

### Application of Nomogram

**Figure 3:**
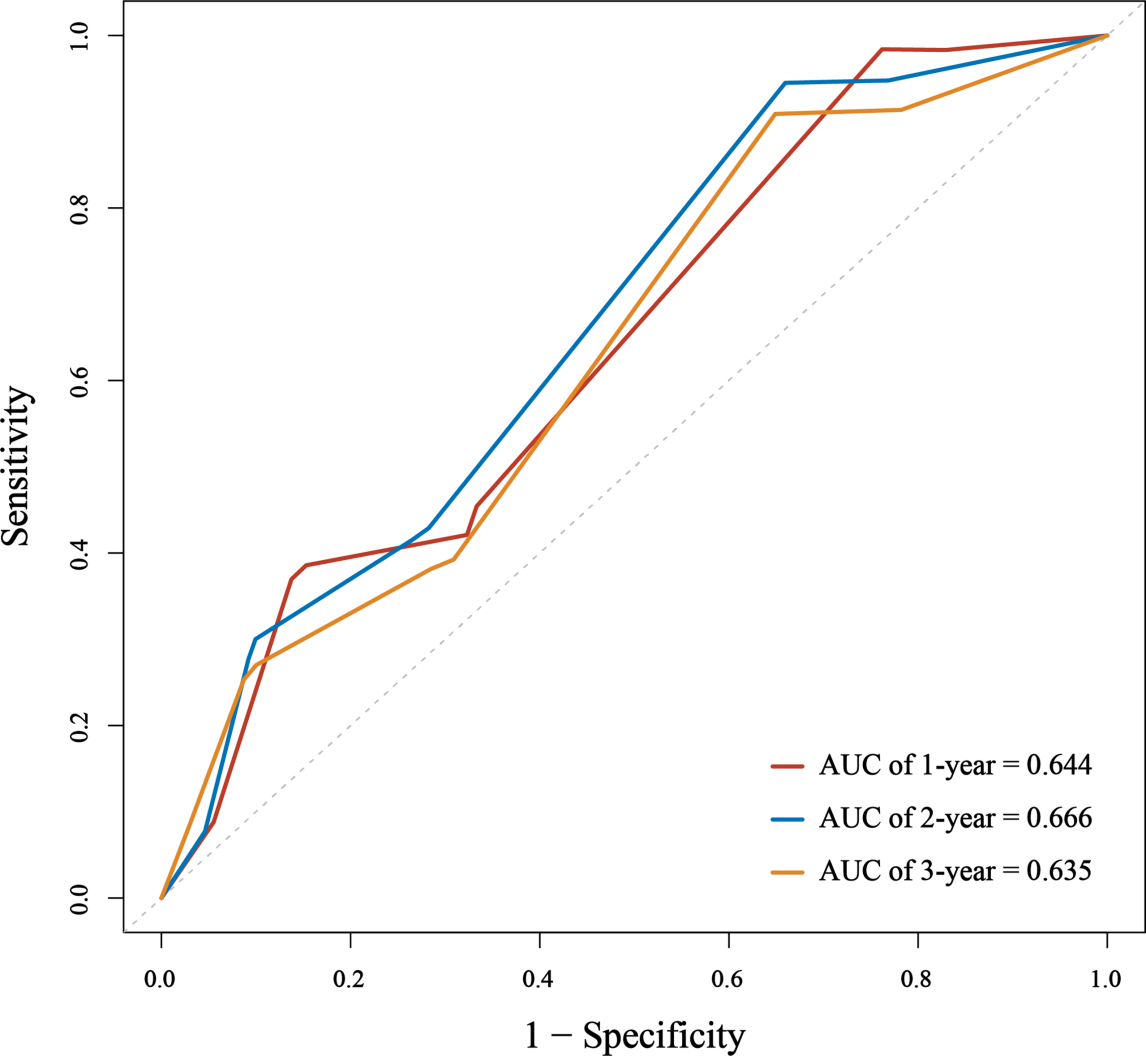
The area under the receiver operating characteristic (ROC) curve (AUC) of the nomogram to predict 1-, 2-, and 3-year overall survival (OS) for small cell lung cancer (SCLC) patients.

**Figure 4:**
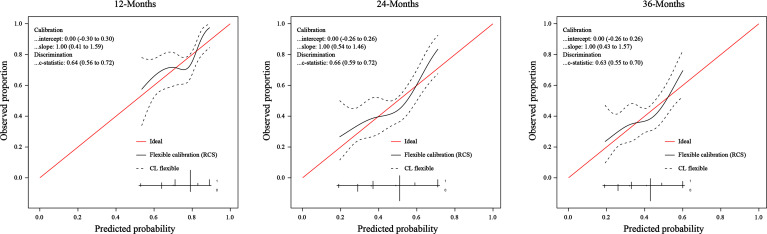
The calibration curve of the nomogram for predicting SCLC patients’ 1-, 2-, and 3- year survival probability.

We assessed the predictive performance of the nomogram in terms of discrimination and calibration. ROC-curve analysis showed that the AUCs of 1-, 2-, and 3-year OS rates were 0.644, 0.666, and 0.635 (**Figure 3**). We constructed a calibration plot based on the observed probability of a 1-, 2-, and 3-year OS against the predicted 1-, 2-, and 3-year OS from the nomogram. In calibration curve analyses, the nomogram showed a nearly ideal curve for the 1-, 2-, and 3-year OS of LS-SCLC patients, with a slope of 1.00 (0.41-1.59), 1.00 (0.54-1.46), and 1.00 (0.43-1.57), respectively (**Figure 4**).

## Discussion

In this study, we have conducted an external application of the Qi’s nomogram to verify the predictive value of Risk and assess the nomogram’s broad applicability. The results showed that Risk could be used as an independent predictor to distinguish prognosis in LS-SCLC patients. The nomogram showed a moderate-to-good performance in another hospital center with different inpatient characteristics.

Several studies have highlighted the value of system immune-inflammation and nutritional parameters in the prognosis prediction for SCLC patients (6, 11–13). Qi et al. formulated a prognostic scoring system called Risk by integrating multiple inflammatory factors, which could more genuinely reflect the complex immune state of the human body compared to a single factor (10). However, due to the different laboratory instruments and reference values applied in the two hospitals, it’s inappropriate to copy the formula in the original article. In this study, we recalculated the cutoff value of PLT, LYM, NLR, PLR, ALB, SII, and reconstructed a Risk using the original methods. The results concluded that the Risk allows distinguishing two groups of patients with different OS, independently from other known prognostic factors, using a threshold of 0.098. The forest plot showed Risk remained a strong predictor of survival in almost all subgroups. The differences in the female and no-smoking subgroups were not statistically significant, which may be due to the small sample size. Following the initial results, Risk was a negative prognostic indicator, and high Risk was associated with an unfavorable prognosis in our cohort.

We found significant differences in clinicopathological features and treatment methods when comparing the baseline characteristics between the external application cohort and the Qi’s cohort. The median OS of patients in our study was shorter than that described in the Qi cohort (22.2 vs. 25.7month). The proportion of receiving PCI in our cohort was significantly lower than that in Qi’s cohort. Treatment choices in the two centers varied dramatically, with almost the opposite ratio. There were also between-group differences in other variables, such as gender, smoke, T stage, and N stage. Various factors, including the economic level, medical resources, public health policies, social habits, and environmental risks, may cause the differences. However, this is the inherent character of real-world population data.

A great deal of research is being devoted to developing new prediction models (12, 14, 15), and external application is lacking. Even if a model is well performed in the developing cohort, its performance deteriorates when applied to a different population, partly because of the potential overfitting and the shift of patient distribution (demographics, clinicopathological characteristics, treatment methods) (16, 17). Inappropriate clinical treatment decisions might be made based on an inaccurate or incorrect prediction model. Independent external application is therefore essential to mitigate institutional bias and ensure the accuracy and reliability of the prediction model before clinical use. Suppose we could further extend the model’s excellent performance to a new cohort from a different center. In that case, the application value of the model in the real world will be significant.

Data obtained in our study indicate a moderate-to-good predictive ability despite the differences mentioned earlier between cohorts of patients. Due to the short follow-up time (20.6months), we evaluated the performance of the nomogram by predicting 1-, 2-, and 3-year OS rates, rather than 1-, 3-, and 5-year OS rates in the original study. We assessed the predictive ability in terms of discrimination and calibration. The discrimination ability of the nomogram in the external application cohort, as measured by the AUC, showed a slight decrease compared to that in the original study. The AUC values of 1-, 2-, and 3-year OS rates were 0.644, 0.666, and 0.635 in our cohort, and the AUC values of 1-, 3-, and 5-year OS rates were 0.717, 0.735, and 0.719 in the original cohort. It’s not surprising that we observed a performance decline in this study. Compared to the original cohort, significantly different baseline characteristics and treatment methods of the external application cohort would affect the nomogram’s discriminative performance. Patients in our cohort were more often male, smokers, and had more advanced T and N stages. Our patients received less PCI and more sequential chemoradiotherapy. One controversy in the original nomogram was that sequential chemoradiotherapy was a favorable factor in the prognosis of patients. However, in our study, it was associated with poor OS. Yet now, the additional benefit of sequential chemoradiotherapy remains unclear for LS-SCLC patients. The above reasons may cause the reduced discrimination of the nomogram in our cohort. Though the discrimination of the nomogram is a little lower than that in the original study, it still had a good predictive ability. The calibration plot of the nomogram in the external application cohort showed a good correlation between the prediction and actual observation for 1-, 2-, and 3-year OS rates, with a calibration slope and intercept of 1.00 and 0.

The nomogram is easily implemented in clinical practice to estimate individualized risk by entering three factors: PCI (Yes, No), Treatment (sequential chemoradiotherapy, concurrent chemoradiotherapy), and Risk (high, low). Our external application study results indicated the nomogram could achieve modest discrimination and ideal calibration. It may be a readily valuable tool to predict LS-SCLC prognosis.

A major limitation in this study was that the median follow-up time in our cohort was 20.6 months, shorter than the 55.9 months in the original cohort. So we did not analyze the 5-year OS prediction due to data limitations. Secondly, the strict selection criteria limited the external application cohort. Last, this was a single-center retrospective study; more external application from other centers with diverse inpatients is needed to assess the applicability of nomograms in the real world.

## Conclusion

The nomogram proposed by Qi et al. was successfully applied in a different population, as it showed clinical meaningful discrimination and accuracy. The Risk could be a strong serum marker in the prognosis prediction of LS-SCLC. Further application in other centers is needed to test the clinical utility of the nomogram.

## Data Availability Statement

The raw data supporting the conclusions of this article will be made available by the authors, without undue reservation.

## Ethics Statement

The studies involving human participants were reviewed and approved by Ethics committee of Shanxi Provincial Cancer hospital (No. 202102). Written informed consent for participation was not required for this study in accordance with the national legislation and the institutional requirements.

## Author Contributions

LW, QH, JL, JC, and SX contributed to conception and design of the study. QH, NY, BS, and LW organized the database. JC and QH performed the statistical analysis. LW wrote the first draft of the manuscript. JC and SX reviewed and edited the manuscript. JL, YL, XC, and HL validated the manuscript. All authors contributed to manuscript revision, read, and approved the submitted version.

## Funding

This work was supported by the Key Research and Development (R&D) Projects of Shanxi Province [grant number 201803D31174] and the Fund Program for the Scientific Activities of Selected Returned Overseas Professionals in Shanxi Province (Department of Resource and Social Security of Shanxi Province No. [2019]1176).

## Conflict of Interest

The authors declare that the research was conducted in the absence of any commercial or financial relationships that could be construed as a potential conflict of interest.

## Publisher’s Note

All claims expressed in this article are solely those of the authors and do not necessarily represent those of their affiliated organizations, or those of the publisher, the editors and the reviewers. Any product that may be evaluated in this article, or claim that may be made by its manufacturer, is not guaranteed or endorsed by the publisher.

## References

[1] Feng R Zong Y Cao S Xu R. Current Cancer Situation in China: Good or Bad News From The 2018 Global Cancer Statistics? Cancer Commun (London England) (2019) 39(1):22. doi: 10.1186/s40880-019-0368-6 PMC6487510 31030667

[2] Siegel R Miller K Jemal A. Cancer Statistics, 2020. CA Cancer J Clin (2020) 70(1):7–30. doi: 10.3322/caac.21590 31912902

[3] Bogart J Waqar S Mix M. Radiation and Systemic Therapy for Limited-Stage Small-Cell Lung Cancer. J Clin Oncol Off J Am Soc Clin Oncol (2022) 40(6):661–70. doi: 10.1200/jco.21.01639 PMC10476774 34985935

[4] Saltos A Shafique M Chiappori A. Update on the Biology, Management, and Treatment of Small Cell Lung Cancer (Sclc). Front Oncol (2020) 10:1074. doi: 10.3389/fonc.2020.01074 32766139 PMC7378389

[5] Liu S Reck M Mansfield A Mok T Scherpereel A Reinmuth N. Updated Overall Survival and Pd-L1 Subgroup Analysis of Patients With Extensive-Stage Small-Cell Lung Cancer Treated With Atezolizumab, Carboplatin, and Etoposide (Impower133). J Clin Oncol Off J Am Soc Clin Oncol (2021) 39(6):619–30. doi: 10.1200/jco.20.01055 PMC8078320 33439693

[6] Galvano A Peri M Guarini A Castiglia M Grassadonia A De Tursi M. Analysis of Systemic Inflammatory Biomarkers in Neuroendocrine Carcinomas of the Lung: Prognostic and Predictive Significance of Nlr, Ldh, Ali, and Lipi Score. Ther Adv Med Oncol (2020) 12:1758835920942378. doi: 10.1177/1758835920942378 32849916 PMC7425322

[7] Wang Y Zhou Y Zhou K Li J Che G. Prognostic Value of Pre-Treatment Red Blood Cell Distribution Width in Lung Cancer: A Meta-Analysis. Biomarkers (2020) 25(3):241–7. doi: 10.1080/1354750x.2020.1731763 32064949

[8] Van Berckelaer C Vermeiren I Vercauteren L Rypens C Oner G Trinh X. The Evolution and Prognostic Role of Tumour-Infiltrating Lymphocytes and Peripheral Blood-Based Biomarkers in Inflammatory Breast Cancer Patients Treated With Neoadjuvant Chemotherapy. Cancers (Basel) (2021) 13(18):4656. doi: 10.3390/cancers13184656 34572883 PMC8471511

[9] Hong H Fang X Huang H Wang Z Lin T Yao H. The Derived Neutrophil-To-Lymphocyte Ratio Is an Independent Prognostic Factor in Patients With Angioimmunoblastic T-Cell Lymphoma. Br J Haematol (2020) 189(5):908–12. doi: 10.1111/bjh.16447 32103494

[10] Qi J Zhang J Ge X Wang X Xu L Liu N. The Addition of Peripheral Blood Inflammatory Indexes to Nomogram Improves the Predictive Accuracy of Survival in Limited-Stage Small Cell Lung Cancer Patients. Front Oncol (2021) 11:713014. doi: 10.3389/fonc.2021.713014 34692490 PMC8531548

[11] Chen C Yang H Cai D Xiang L Fang W Wang R. Preoperative Peripheral Blood Neutrophil-To-Lymphocyte Ratios (Nlr) and Platelet-To-Lymphocyte Ratio (Plr) Related Nomograms Predict the Survival of Patients With Limited-Stage Small-Cell Lung Cancer. Trans Lung Cancer Res (2021) 10(2):866–77. doi: 10.21037/tlcr-20-997 PMC7947425 33718028

[12] Xie D Marks R Zhang M Jiang G Jatoi A Garces YI. Nomograms Predict Overall Survival for Patients With Small-Cell Lung Cancer Incorporating Pretreatment Peripheral Blood Markers. J Thorac Oncol (2015) 10(8):1213–20. doi: 10.1097/jto.0000000000000585 26200277

[13] Suzuki R Wei X Allen P Cox J Komaki R Lin S. Prognostic Significance of Total Lymphocyte Count, Neutrophil-To-Lymphocyte Ratio, and Platelet-To-Lymphocyte Ratio in Limited-Stage Small-Cell Lung Cancer. Clin Lung Cancer (2019) 20(2):117–23. doi: 10.1016/j.cllc.2018.11.013 30611672

[14] Li B Jiang C Wang R Zou B Xie P Li W. <P>Prognostic Value of a Nomogram Based on the Dynamic Albumin-To-Alkaline Phosphatase Ratio for Patients With Extensive-Stage Small-Cell Lung Cancer</P>. Onco Targets Ther (2020) 13:9043–57. doi: 10.2147/ott.s262084 PMC7494229 32982294

[15] Xiao H Zhang B Liao X Yan S Zhu S Zhou F. Development and Validation of Two Prognostic Nomograms for Predicting Survival in Patients With Non-Small Cell and Small Cell Lung Cancer. Oncotarget (2017) 8(38):64303–16. doi: 10.18632/oncotarget.19791 PMC5610004 28969072

[16] Steyerberg EW Vergouwe Y. Towards Better Clinical Prediction Models: Seven Steps for Development and an Abcd for Validation. Eur Heart J (2014) 35(29):1925–31. doi: 10.1093/eurheartj/ehu207 PMC4155437 24898551

[17] Ramspek C Jager K Dekker F Zoccali C van Diepen M. External Validation of Prognostic Models: What, Why, How, When and Where? Clin Kidney J (2021) 14(1):49–58. doi: 10.1093/ckj/sfaa188 33564405 PMC7857818

